# Association of plasma brain-derived neurotrophic factor with Alzheimer’s disease and its influencing factors in Chinese elderly population

**DOI:** 10.3389/fnagi.2022.987244

**Published:** 2022-11-08

**Authors:** Fuqiang Qian, Jian Liu, Hongyu Yang, Haohao Zhu, Zhiqiang Wang, Yue Wu, Zaohuo Cheng

**Affiliations:** ^1^The Affiliated Wuxi Mental Health Center of Jiangnan University, Wuxi Central Rehabilitation Hospital, Wuxi, China; ^2^Hangzhou Seventh People’s Hospital, Hangzhou, China; ^3^Shanghai Mental Health Center, Shanghai, China

**Keywords:** Alzheimer’s disease, biomarker, BDNF, influencing factors, elderly population

## Abstract

**Objective:**

To explore the association of plasma brain-derived neurotrophic factor (BDNF) levels with Alzheimer’s disease and its influencing factors.

**Materials and methods:**

A total of 1,615 participants were included in the present study. Among all subjects, 660 were cognitive normal controls (CNCs), 571 were mild cognitive impairment (MCI) patients, and 384 were dementia with Alzheimer’s type (DAT) patients. BDNF in blood samples collected from these subjects was analyzed via the Luminex assay. Additionally, DNA extraction and APOE4 genotyping were performed on leukocytes using a blood genotyping DNA extraction kit. All data were processed with SPSS 20.0 software. Analysis of variance (ANOVA) or analysis of covariance (ANCOVA) was used to compare differences among groups on plasma BDNF. Pearson and Spearman correlation analysis examined the correlation between BDNF and cognitive impairment, and linear regression analysis examined the comprehensive effects of diagnosis, gender, age, education, and sample source on BDNF.

**Results:**

BDNF levels in DAT patients were higher than those in CNC and MCI patients (*P* < 0.01). BDNF levels were significantly correlated with CDR, MMSE, and clinical diagnosis (*P* < 0.001). Age, education, occupation, and sample source had significant effects on BDNF differences among the CNC, MCI, and DAT groups (*P* < 0.001). BDNF first decreased and then increased with cognitive impairment in the ApoE4-negative group (*P* < 0.05).

**Conclusion:**

Plasma BDNF levels decreased in the MCI stage and increased in the dementia stage and were affected by age, education, occupation, and sample source. Unless the effects of sample heterogeneity and methodological differences can be excluded, plasma BDNF is difficult to become a biomarker for the early screening and diagnosis of AD.

## Introduction

With the growth and longevity of the elderly population, Alzheimer’s disease (AD) has become a global human health problem. The Seventh China National Census reported that there were 264 million elderly people over 60 years old in China in 2020. By a prevalence of 4%, it is estimated that there will be more than 10 million AD patients in 2020, 20 million in 2035, and 40 million in 2060 (Cheng, 2020; Zhao and Li, 2020). AD is a chronic progressive and irreversible neurodegenerative disease (Alzheimer Association, 2017; Knopman et al., 2021). When AD progresses to the dementia stage, there are no disease-modifying drugs to prevent or reverse the disease process (Yiannopoulou and Papageorgiou, 2020). Therefore, early diagnosis and intervention of AD is particularly important. Many researchers are committed to looking for early diagnosis markers of AD, and brain-derived neurotrophic factor (BDNF) is one of the targets (Cheng et al., 2018; Rehiman et al., 2020).

Brain-derived neurotrophic factor plays an important role in brain development, neuroplasticity, and neuronal survival and its effects on neuronal transmission in the hippocampus, cerebral cortex, and basal forebrain are associated with learning and memory processes in the mature brain (Popova and Naumenko, 2019). Growing evidence indicates that changes in cerebral BDNF levels and the BDNF-TrkB signaling pathway may be involved in the etiopathogenesis of AD and that serum and brain BDNF levels are correlated. This evidence suggests that blood BDNF could be used as a biomarker for AD diagnosis, prognosis, and treatment monitoring (Ng et al., 2019; Marta et al., 2020; Mori et al., 2021).

Several recent meta-analyses analyzed the association between BDNF and AD cognitive impairment and found that the results are inconsistent or even contradictory, and higher, lower, or similar levels of circulating BDNF have all been reported in AD or MCI patients compared to healthy controls (Qin et al., 2017; Ng et al., 2019; Xie et al., 2020; Ma et al., 2022). For example, Kim et al. (2017) showed that blood BDNF levels seem to be increased in early AD and decreased in AD patients with low MMSE scores (Kim et al., 2017). Ng et al. (2019) reported that blood BDNF did not change in the MCI stage and only decreased in the late stage of AD. Not only are the conclusions of the difference in BDNF between AD patients and normal controls inconsistent, but there are also great differences in BDNF levels among different studies. Some studies reported that BDNF levels were very low (<5 pg/ml) (Nascimento et al., 2014; Wang et al., 2015), while others reported that BDNF levels were very high (>50 ng/ml) (Liu et al., 2015; Zhang et al., 2019).

These contradictory results may be related to sample heterogeneity (age, gender, education, condition, comorbidity, and medication) and methodological differences (cognitive rating tools and diagnostic criteria, sample type, blood processing, storage duration, etc.) (Balietti et al., 2018); for instance, blood BDNF decreased with age or weight in elderly individuals (Glud et al., 2019), increased in the early AD stage and decreased in the late stage (Laske et al., 2006). It is very important to study and control the influence of these factors on the association between BDNF levels and cognitive impairment, and it is more important to consider the possible influence of various factors in the interpretation of results. This study investigated the association of plasma BDNF levels with cognitive impairment and its influencing factors.

## Materials and methods

### Participants

This study recruited adult and elderly volunteers over 50 years old from communities and mental health hospitals in four cities with different levels (Shanghai, Hangzhou, Wuxi, Jiangyi). The unified training, containing the purpose, content, and methods, was organized to ensure the comparability of the procedures. All subjects received clinical interviews and examinations, relevant cognitive evaluations, necessary laboratory or imaging examinations, and overnight fasting venous blood was collected. Based on interviews, assessments and examinations, 1,615 valid samples were obtained after excluding serious physical, neurological and mental disorders that may cause cognitive impairment (including uncontrolled hypertension, diabetes, Parkinson’s disease, cerebrovascular disease, hypothyroidism, schizophrenia, depression, etc.), including 230 persons in Shanghai, 248 in Hangzhou, 937 in Wuxi, and 200 in Jiangyi. According to the NINCDS-ADRDA criteria (McKhann et al., 1984) for dementia with Alzheimer’s type (DAT), DSM-5 criteria (American Psychiatric Association, 2013) for neurocognitive impairment and Petersen criteria (Petersen et al., 1999) for mild cognitive impairment (MCI), 1,615 participants were clinically diagnosed into three groups: 660 cognitive normal controls (CNCs), 571 MCI patients and 384 DAT patients, of which 218 DAT patients were from outpatients and inpatients in mental health hospitals, and other DAT patients and CNC and MCI subjects were from community volunteers. Sample sources and basic information are shown in Table 1.

**TABLE 1:** Sample source and basic data.

		Total sample	Wuxi	Jiangyi	Hangzhou	Shanghai	χ^2^ or F	*P*
Gender (male/female)		678/937	432/505	86/114	87/161	73/157	21.380	<0.001
Age (years)		70.09 ± 9.33	67.89 ± 7.83	67.46 ± 8.16	77.98 ± 9.40	78.53 ± 7.03	189.968	<0.001
Education (years)		7.96 ± 4.07	9.41 ± 3.12	7.61 ± 2.71	5.58 ± 4.57	4.88 ± 4.89	140.581	<0.001
Diagnosis (HC/MCI/DAT)		660/571/384	476/281/180	33/139/28	50/49/149	101/102/27	341.868	<0.001
CDR-GS (score)		0.61 ± 0.70	0.55 ± 0.68	0.60 ± 0.63	0.99 ± 0.84	0.44 ± 0.54	32.576	<0.001
BDNF (pg/mL)	Mean ± SD Ln(X + 1)	10555.7 ± 12135.1 8.55 ± 1.40	10373.5 ± 9646.3 8.82 ± 1.05	3632.0 ± 5605.6 7.51 ± 1.29	24976.2 ± 16152.4 9.79 ± 1.17	1769.4 ± 2026.5 7.01 ± 0.97	267.081 338.724	<0.001 <0.001

This study was approved by the ethics committee of Wuxi Mental Health Center, the ethics committee of Shanghai Mental Health Center and the ethics committee of Hangzhou Seventh People’s Hospital. According to the Declaration of Helsinki, all subjects or their caregivers were informed of the procedure and signed informed consent was obtained before participating in the study.

### Clinical interview and examination

Clinical interviews and examinations included four primary sections. (A) Social demographic data: name, gender, age, nationality, marriage, education, occupation, family structure, economic status, smoking and drinking habits, outdoor activities, etc.; (B) Medical history and mental examination: memory and cognitive impairment, mental status examination, family history, past medical history and individual medication; (C) physical examination: general examination, such as heart rate, blood pressure, height, weight, vision, hearing, hair color, and facial plaques, etc., emphasis on neurological examination, such as sensory symmetry, motor function, muscle strength, muscle tone, language function, gait and balance, tremor. (D) Necessary auxiliary examination: ECG, EEG, brain CT, blood biochemistry tests, etc. Only the corresponding author (ZC) could obtain the details of each parameter of the patients. The cognitive test interviewers were only responsible for the scale assessment.

### Psychological and neurocognitive assessment

Assessments involved three important aspects: subjective cognitive impairment screening, objective cognitive impairment assessment, and related mental rating. Subjective cognitive impairment of elderly volunteers in the community was screened using Brief Elderly Cognitive Screening Questionnaire Screening (BECSI) (Wu et al., 2016), with a total score of more than four points indicating subjective cognitive impairment. Overall objective cognitive impairments were assessed by the Clinical Dementia Rating (CDR) (Academy of Cognitive Disorder of China, 2018), Alzheimer’s Dementia Assessment Scale-cognitive subscale (ADAS-cog) (Wang et al., 2000), and/or Mini Mental State Examination (MMSE) (Li et al., 1988). The cognitive function level of each subject was determined by the cutoff score of each scale (CDR: 0 points for normal cognition, 0.5 for mild cognitive impairment, ≥1 for severe cognitive impairment; ADAS-cog: 0–9 for normal cognition, 10–15 for mild cognitive impairment, ≥ 16 points for severe cognitive impairment, or MMSE: 28–30 for normal cognition, 20–27 for mild cognitive impairment, <20 for severe cognitive impairment). Other related mental ratings included the Activity of Daily Living Scale (ADL) (Lawton and Brody, 1969), Hachinski Ischemic Scale (HIS) (Fan, 1990) and Hamilton Depression Scale (HAMD) (Zhao and Zheng, 1992).

### Plasma brain-derived neurotrophic factor assays and APOE genotyping

Blood samples were collected in anticoagulant tubes with 2% EDTA in the morning after an overnight fast. Blood samples were centrifuged at 3,000 rpm (1,000 × *g*) for 30 min at 4°C. Plasma and leukocytes were collected in plastic vials and stored at −80°C for further analyses. Only the corresponding author (ZC) could obtain the details of each parameter of the patients. The blood lab analysis staff was blinded to other results, since the patients were represented by numbers.

#### Luminex assays

Protein detection was entrusted to Nanjing University of Technology (laboratory certificated Millipore Shanghai Trading Co., Ltd., based on Milliplex technology). The Luminex kit (Milliplex Catalog ID. HNDG3MAG-36K-04. Neu) were obtained from Millipore (Billerica, MA, USA), and assays were performed according to the manufacturer’s instructions to determine the plasma levels of multiple proteins, including BDNF (pg/mL). Properly diluted plasma samples were incubated with the antibody-coupled microspheres and then with biotinylated detection antibody before the addition of streptavidin-phycoerythrin. The captured bead complexes were measured with a FLEXMAP 3D system (Luminex Corporation, Austin, TX, USA) using the following instrument settings (events/bead, 50; sample size, 50 μL; discriminator gate, 8,000–15,000). The raw data (mean fluorescence intensity) were collected and further processed to calculate the protein concentration. Before statistical analysis, we examined the performance of each assay using quality checks (QC). Median fluorescent intensity (MFI) was measured using xPONENT 5.1 (Luminex Corporation) and exported into Milliplex Analyst 5.1 (VigeneTech, Carlisle, MA, USA) for estimation of protein concentrations using a five-parameter logistic fit. Briefly, all analysts that passed QC checks based on the following four criteria (standard curve linearity, intra-assay coefficient of variation, interassay coefficient of variation for reference sample, and percentage of missing data) were taken forward for further analysis.

#### DNA extraction and *APOE4* genotyping

DNA extraction and *APOE4* genotyping were performed by Wuxi Biowing Applied Biotechnology Co., Ltd. DNA was extracted from leukocytes using a blood genotyping DNA extraction kit (Tiangen Biotech, Beijing, China), and the *APOE4* genotype was analyzed using polymerase chain reaction restriction fragment length polymorphism (PCR-RFLP). According to the methods reported by Hixson and Vernier (1990), *APOE4* gene primers were designed and synthesized, and PCR amplification, enzyme digestion, and *APOE4* genotyping were performed. The three alleles were grouped into six genotypes, which were *APOE4* negative (E2/E2, E3/E3, E2/E3) and *APOE4* positive (E2/E4, E3/E4, E4/E4).

### Data processing and statistical analysis

All data were processed with SPSS 20.0 software. Logarithmic transformation [Ln(x + 1)] of BDNF data was performed to approximate a normal distribution. Pearson’s chi-square test was used to compare differences among groups on gender, marriage, family, occupation, and *APOE4 genotypes*. The F test was used to compare differences among groups for age, years of education, blood pressure, body mass index, psychological test scores and biochemical test results. Analysis of variance (ANOVA) or analysis of covariance (ANCOVA) was used to compare differences among groups on plasma BDNF. Pearson and Spearman correlation analysis examined the correlation between BDNF and cognitive impairment, and linear regression analysis examined the comprehensive effects of diagnosis, gender, age, education, and sample source on BDNF.

## Results

### Comparison of demographic, clinical and laboratory data among the three groups

The differences in the demographic, clinical and laboratory data among the CNC, MCI, and DAT groups are shown in Table 2. There were significant differences among the three groups in demographic data, such as sex, age, education, marital status, family type, and previous occupation (*P* < 0.01). There were no significant differences in body mass index (BMI), systolic blood pressure (SBP) or diastolic blood pressure (DBP) among the groups (*P* > 0.05). There were no significant differences among the three groups in biochemical indexes such as fasting blood glucose (FBG), triglycerides, thyroid stimulating hormone (TSH), free T4, folic acid and vitamin B12 (*P* > 0.05), but there was a significant difference in laboratory indexes such as *APOE* genotype, total protein, albumin, total cholesterol, high density lipoprotein (HDL), low density lipoprotein (LDL), free T3 and BDNF (*P* < 0.05). There were significant differences in cognitive evaluation scores such as ADAS-cog, MMSE, CDR-GS, and ADL (*P* < 0.001).

**TABLE 2:** Demographic, clinical and laboratory data and cognitive scores of the three groups.

		Total sample (*n* = 1615)	CNC (*n* = 660)	MCI (*n* = 571)	DAT (*n* = 384)	χ^2^ or *F*-value	*P*-value
Gender (Male/Female)		678/937	306/354	229/342	143/241	9.574	0.008
Age (years)		70.09 ± 9.33	68.21 ± 8.26	69.91 ± 8.74	77.00 ± 9.19	130.867	<0.001
Education (years)		7.96 ± 4.07	9.49 ± 3.59	7.44 ± 3.73	6.11 ± 4.37	101.896	<0.001
Marriage (in marriage/other)		1383/232	598/62	490/81	255/129	21.967	<0.001
Family (Big family/Couple/Others)		568/864/183	243/385/32	218/315/38	107/164/113	131.930	<0.001
Occupation (Physical/Technical/Intellectual)		197/1072/346	43/416/201	134/363/74	20/293/71	116.592	<0.001
BMI (kg/m^2^)		23.85 ± 3.11	23.74 ± 2.92	24.07 ± 3.29	23.51 ± 3.25	1.848	0.156
SBP (mm/Hg)		136.25 ± 16.76	135.87 ± 16.47	135.91 ± 16.40	139.76 ± 19.45	2.233	0.108
DBP (mm/Hg)		82.23 ± 9.45	82.78 ± 9.28	81.52 ± 9.44	82.29 ± 10.28	1.955	0.142
*APOE4* (Positive/Negative)		176/795	80/387	59/330	37/78	17.899	<0.001
Total protein (g/L)		75.27 ± 4.91	75.68 ± 4.93	74.98 ± 4.72	74.31 ± 5.41	4.292	0.014
Albumin (g/L)		46.20 ± 2.96	46.60 ± 2.82	45.95 ± 2.99	45.06 ± 3.18	13.236	<0.001
FBG (mmol/L)		6.14 ± 1.62	6.11 ± 1.62	6.16 ± 1.68	6.21 ± 1.42	0.212	0.809
Triglyceride (mmol/L)		1.66 ± 1.31	1.66 ± 1.08	1.63 ± 1.30	1.81 ± 2.19	0.737	0.473
Total cholesterol (mmol/L)		5.12 ± 1.02	5.17 ± 0.97	5.15 ± 1.09	4.81 ± 0.90	4.981	0.007
HDL (mmol/L)		1.23 ± 0.36	1.19 ± 0.31	1.29 ± 0.41	1.22 ± 0.33	8.533	<0.001
LDL (mmol/L)		2.95 ± 0.74	3.02 ± 0.74	2.93 ± 0.75	2.64 ± 0.65	10.307	<0.001
TSH (mIU/L)		2.33 ± 2.08	2.24 ± 1.65	2.36 ± 1.82	2.75 ± 4.54	1.862	0.156
FreeT3 (pmol/L)		4.77 ± 1.55	4.98 ± 1.39	4.42 ± 1.33	4.92 ± 2.83	13.707	<0.001
FreeT4 (pmol/L)		15.67 ± 3.84	15.87 ± 3.72	15.46 ± 3.97	15.32 ± 4.01	1.408	0.245
Folic acid (nmol/L)		44.63 ± 77.24	50.25 ± 90.47	37.20 ± 53.83	41.11 ± 69.30	2.839	0.059
Vitamin B12 (pmol/L)		350.01 ± 198.01	356.73 ± 202.76	345.94 ± 189.65	327.82 ± 201.96	0.902	0.406
CDR-GS (score)		0.61 ± 0.70	0.18 ± 0.28	0.50 ± 0.22	1.50 ± 0.86	573.035	<0.001
ADAS-cog (score)		9.38 ± 6.22 (*n* = 996)	6.16 ± 2.56 (*n* = 508)	11.48 ± 5.26 (*n* = 419)	20.25 ± 11.05 (*n* = 69)	326.625	<0.001
MMSE (score)		19.46 ± 8.88 (*n* = 859)	27.40 ± 3.35 (*n* = 210)	23.33 ± 4.29 (*n* = 318)	10.72 ± 7.01 (*n* = 331)	754.315	<0.001
ADL (score)		18.48 ± 13.04 (*n* = 1308)	13.67 ± 5.74 (*n* = 557)	14.85 ± 6.68 (*n* = 468)	33.95 ± 18.48 (*n* = 283)	417.864	<0.001
BDNF (pg/mL)	Mean ± SD Ln(X + 1)	10555.7 ± 12135.1 8.55 ± 1.40	10101.6 ± 11748.3 8.50 ± 1.41	8556.3 ± 10255.5 8.32 ± 1.39	14309.1 ± 14356.8 8.98 ± 1.29	27.452 27.302	<0.001 <0.001

### Relationship between plasma brain-derived neurotrophic factor and cognitive function

**FIGURE 1:**
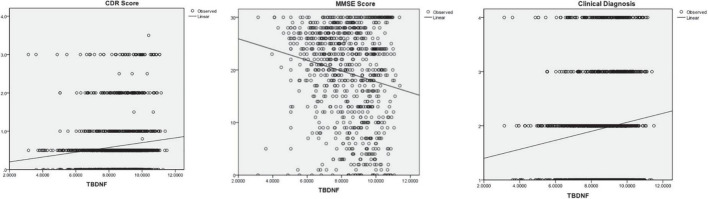
The correlations between Brain-Derived Neurotrophic Factor (BNDF) and Clinical Dementia Rating (CDR), Mini Mental State Examination (MMSE), and clinical diagnosis.

The subjects were divided into four groups (CNC, MCI, mild dementia, and moderate-severe dementia) according to the CDR, MMSE or clinical diagnosis. The plasma BDNF differences among groups were compared by ANCOVA (age and education as covariates), and the correlation between BDNF and cognitive function was calculated by Pearson and Spearman correlation analysis. The results are shown in Table 3 and Figure 1. There was a significant difference in BDNF among the four groups (*P* < 0.01). BDNF levels in dementia patients were higher than those in CNC and MCI patients (*P* < 0.01), and there was no significant difference in BDNF between the CNC and MCI groups. BDNF levels were significantly correlated with CDR, MMSE, and clinical diagnosis (*P* < 0.001). BDNF was positively correlated with CDR scores and clinical diagnosis, and negatively correlated with MMSE score.

**TABLE 3:** Association between BDNF and cognitive function.

	CNC	MCI	Mild dementia	Moderate-severe dementia	ANCOVA Bonferroni test	Pearson correlation	Spearman correlation
CDR group	8.35 ± 1.39 (*n* = 486)	8.53 ± 1.40 (*n* = 778)	8.85 ± 1.28 (*n* = 164)	8.89 ± 1.38 (*n* = 187)	*F* = 17.783, *P* = 0.000 CNC = MCI < Mild = severe	*r* = 0.124 *P* ≤ 0.001	*r_*s*_* = 0.124 *P* ≤ 0.001
MMSE group	8.14 ± 1.70 (*n* = 236)	8.01 ± 1.57 (*n* = 269)	8.57 ± 1.46 (*n* = 182)	8.86 ± 1.45 (*n* = 172)	*F* = 12.632, *P* = 0.000 MCI = CNC < Mild = severe	*r* = −0.183 *P* ≤ 0.001	*r*_*s*_ = 0.183 *P* ≤ 0.001
Clinical diagnosis	8.50 ± 1.41 (*n* = 660)	8.32 ± 1.39 (*n* = 571)	9.08 ± 1.19 (*n* = 198)	8.88 ± 1.38 (*n* = 186)	*F* = 26.667, *P* = 0.000 MCI = CNC < Mild = severe		*r*_*s*_ = 0.117 *P* ≤ 0.001

### Factors influencing the association of brain-derived neurotrophic factor with cognitive impairment

The results of ANCOVA and ANOVA are shown in Table 4. Age, education, occupation and sample source had significant effects on BDNF differences among the CNC, MCI, and DAT groups (*P* < 0.001), and sex and APOE4 had no significant effect on BDNF differences among the groups (*P* > 0.05). The trend of the BDNF expression was the same in different genders with significant difference (*P* = 0.000), which decreased in MCI and increased in DAT. BDNF first decreased and then increased significantly with the aggravation of cognitive impairment in groups aged 51–64 and 65–74 (*P* < 0.05), and BDNF increased gradually with cognitive impairment in the group over 75 years old (*P* < 0.05). BDNF increased gradually with cognitive impairment in the group with 0–6 years of education (*P* < 0.05), and there was no significant change in BDNF with cognitive impairment in the other two groups (*P* > 0.05). BDNF first decreased and then increased significantly with cognitive impairment in physical and technical occupation (*P* < 0.05), and BDNF increased gradually with cognitive impairment in intellectual occupation (*P* > 0.05). BDNF increased gradually with cognitive impairment in the Shanghai group (*P* = 0.010), and there was no significant change in cognitive impairment in other districts (*P* > 0.05). BDNF in the male and female groups first decreased and then increased significantly with cognitive impairment (*P* < 0.001). BDNF first decreased and then increased with cognitive impairment in the ApoE4-negative group (*P* < 0.05), and there was no significant change in BDNF with cognitive impairment in the ApoE4-positive group (*P* > 0.05).

**TABLE 4:** Factors influencing the association between plasma BDNF and cognitive impairment.

Relevant factors		CNC(A)	MCI(B)	DAT(C)	ANCOVA	ANOVA	LSD test
Gender	Male	8.44 ± 1.34 (*n* = 306)	8.28 ± 1.38 (*n* = 229)	8.98 ± 1.25 (*n* = 143)	Group: F = 26.998 *P* ≤ 0.001 Gender: *F* = 1.182 *P* = 0.277	*F* = 12.905 *P* ≤ 0.001	C > A = B
	Female	8.55 ± 1.46 (*n* = 354)	8.35 ± 1.41 (*n* = 342)	8.98 ± 1.31 (*n* = 241)		*F* = 14.411 *P* ≤ 0.001	C > A = B
	ANOVA	*F* = 1.089 *P* = 0.297	*F* = 0.427 *P* = 0.514	*F* = 0.000 *P* = 0.995			
Age	51–64 years	8.76 ± 1.28 (*n* = 255)	8.45 ± 1.35 (*n* = 186)	8.92 ± 1.21 (*n* = 47)	Group: *F* = 37.030 *P* ≤ 0.001 Age: *F* = 26.844 *P* = 0.001	*F* = 4.019 *P* = 0.019	C = A > B
	65–74 years	8.68 ± 1.29 (*n* = 255)	8.29 ± 1.38 (*n* = 200)	9.03 ± 1.16 (*n* = 95)		*F* = 11.513 *P* ≤ 0.001	C > A > B
	>75 years	7.75 ± 1.55 (*n* = 150)	8.23 ± 1.45 (*n* = 185)	8.98 ± 1.35 (*n* = 242)		*F* = 36.034 *P* ≤ 0.001	C > B > A
	ANOVA	*F* = 30.269 *P* = 0.000	*F* = 1.247 *P* = 0.288	*F* = 0.129 *P* = 0.879			
Education	0–6 years	8.09 ± 1.54 (*n* = 136)	8.12 ± 1.47 (*n* = 276)	9.05 ± 1.23 (*n* = 247)	Group: *F* = 30.665 *P* ≤ 0.001 Education: *F* = 10.703 *P* = 0.001	*F* = 34.906 *P* ≤ 0.001	C > A = B
	7–9 years	8.62 ± 1.33 (*n* = 277)	8.46 ± 1.31 (*n* = 207)	8.87 ± 1.32 (*n* = 75)		*F* = 2.765 *P* = 0.064	C = A = B
	>10 years	8.58 ± 1.38 (*n* = 247)	8.63 ± 1.23 (*n* = 88)	8.84 ± 1.45 (*n* = 50)		*F* = 0.894 *P* = 0.410	C = A = B
	ANOVA	*F* = 7.539 *P* = 0.001	*F* = 6.135 *P* = 0.002	*F* = 1.047 *P* = 0.352			
Occupation	Physical	8.32 ± 1.24 (*n* = 37)	7.81 ± 1.41 (*n* = 110)	8.74 ± 1.37 (*n* = 15)	Group: *F* = 26.556 *P* ≤ 0.001 Occupation: F = 22.376 *P* ≤ 0.001	*F* = 4.243 *P* = 0.016	C > B, A = B
	Technical	8.88 ± 1.23 (*n* = 352)	8.80 ± 1.22 (*n* = 298)	9.39 ± 1.09 (*n* = 218)		*F* = 17.803 *P* ≤ 0.001	C > A = B
	Intellectual	8.75 ± 1.30 (*n* = 170)	8.98 ± 1.10 (*n* = 61)	9.17 ± 1.21 (*n* = 53)		*F* = 2.648 *P* = 0.073	C > A, A = B
	ANOVA	*F* = 3.530 *P* = 0.030	*F* = 28.161 *P* = 0.000	*F* = 2.859 *P* = 0.059			
Sample sources	Wuxi	8.79 ± 1.10 (*n* = 476)	8.87 ± 1.03 (*n* = 281)	8.84 ± 0.96 (*n* = 180)	Group: *F* = 0.405 *P* = 0.667 Source: *F* = 896.678 *P* ≤ 0.001	*F* = 0.523 *P* = 0.593	A = B = C
	Jiangyi	7.42 ± 1.13 (*n* = 33)	7.56 ± 1.34 (*n* = 139)	7.33 ± 1.21 (*n* = 28)		*F* = 0.486 *P* = 0.616	A = B = C
	Hangzhou	9.80 ± 1.75 (*n* = 50)	9.91 ± 0.60 (*n* = 49)	9.75 ± 1.09 (*n* = 149)		*F* = 0.324 *P* = 0.723	A = B = C
	Shanghai	6.82 ± 0.94 (*n* = 101)	7.09 ± 0.99 (*n* = 102)	7.41 ± 0.90 (*n* = 27)		*F* = 4.278 *P* = 0.010	C > A = B
	ANOVA	*F* = 115.003 *P* = 0.000	*F* = 125.701 *P* = 0.000	*F* = 74.665 *P* = 0.000			
*APOE4*	Negative	8.74 ± 1.15 (*n* = 387)	8.43 ± 1.25 (*n* = 330)	8.90 ± 1.04 (*n* = 78)	Group: *F* = 9.318 *P* ≤ 0.001 APOE: *F* = 40.063 *P* = 0.802	*F* = 8.434 *P* ≤ 0.001	C = A > B
	Positive	8.63 ± 1.27 (*n* = 80)	8.49 ± 1.39 (*n* = 59)	8.91 ± 0.93 (*n* = 37)		*F* = 1.231 *P* = 0.295	C = A = B
		*F* = 0.535 *P* = 0.465	*F* = 0.130 *P* = 0.719	*F* = 0.001 *P* = 0.971			

### The combined effects of related factors on brain-derived neurotrophic factor

The combined effect of diagnosis, gender, age, education, and sample source on plasma BDNF was examined by ANCOVA and linear regression analysis, and detailed results are shown in Table 5. The effects of diagnosis, age and education on plasma BDNF were statistically significant (*P* < 0.01), but the gender effect was not statistically significant (*P* = 0.149), and only 5.1% of the variation could be explained. After adding the sample source, only the sample source and gender effects were statistically significant (*P* < 0.05), the effects of diagnosis, age and education were not statistically significant (*P* > 0.05), and the explained variation reached 37.9%. Linear regression analysis showed that the four factors could explain 3.0% of BDNF variation, of which the effects of diagnosis, age, and education were statistically significant (*P* = 0.000). Adding the sample source explained 38.0% of the BDNF variation; only the effects of sample source and sex were statistically significant (*P* < 0.01), and the effects of diagnosis, age and education were not statistically significant (*P* > 0.05).

**TABLE 5:** The comprehensive effects of diagnosis, gender, age, education and sample source on brain-derived neurotrophic factor.

Related factors	ANCOVA			Linear regression			
	χ^2^	*F*	*P*	B	β	*t*	*P*
Diagnosis	71.948	38.858	<0.001	0.308	0.173	6.446	<0.001
Gender	3.864	2.087	0.149	0.11	0.039	1.536	0.125
Age	38.854	20.984	<0.001	–0.172	–0.100	–3.817	<0.001
Education	14.019	7.572	0.006	0.167	0.095	3.504	<0.001

	***R*^2^ = 0.053, Adjust *R*^2^ = 0.051**			***R*^2^ = 0.033, Adjust *R*^2^ = 0.030**			
Diagnosis	0.32	0.265	0.767	0.021	0.012	0.533	0.594
Gender	9.503	7.853	0.005	0.16	0.056	2.791	0.005
Age	0.033	0.027	0.869	–0.009	–0.005	–0.252	0.801
Education	0.009	0.008	0.93	0.002	0.001	0.041	0.967
Sample sources	1033.37	853.958	<0.001	0.970	0.615	30.135	<0.001

	*R*^2^ = 0.382, Adjust *R*^2^ = 0.379			*R*^2^ = 0.382, Adjust *R*^2^ = 0.380			

## Discussion

Growing evidence indicates that BDNF is associated with the pathophysiology of AD. However, the association of BDNF levels in the brain or peripheral blood with cognitive impairment in AD is quite complex and influenced by many factors, such as sample heterogeneity and methodological differences (Kim et al., 2017; Qin et al., 2017; Balietti et al., 2018; Ng et al., 2019; Marta et al., 2020; Xie et al., 2020; Girotra et al., 2022). In the present study, the association of plasma BDNF with AD cognitive impairment and influencing factors was investigated in depth based on a large sample of elderly people in the community. It was preliminarily found that plasma BDNF concentrations were related to AD cognitive impairment but not a simple linear relationship. Since there were significant difference between CNC, MCI, and DAT cohorts regarding age, sex, education, and social status, the factors influencing the association between plasma BDNF and cognitive impairment were analyzed separately. The alterations in the plasma BDNF levels of AD depended on the stages or severity of AD and were affected by factors such as age, education or sample source. The main findings of this study are briefly analyzed and discussed as follows.

Many studies have found alterations in blood BDNF levels in patients with AD and MCI, but the results are inconsistent or even contradictory. Most studies observed a decrease in blood BDNF levels (Borba et al., 2016; Siuda et al., 2017; Zhang et al., 2019; Tang et al., 2020), a few reported an increase in BDNF concentrations (Lee et al., 2015; Ng et al., 2021), and other studies reported no significant change in BDNF levels (O’Bryant et al., 2011; Wang et al., 2015). Xie et al. (2020) meta-analysis showed that peripheral blood BDNF decreased gradually with the aggravation of cognitive impairment. Ng et al. (2019) meta-analysis showed that peripheral BDNF levels decreased in patients with AD and could only be detected at the late stage of the dementia spectrum. Qin et al. (2017) suggested that AD or MCI is accompanied by reduced peripheral blood BDNF levels, supporting an association between the decreasing levels of BDNF and the progression of AD.

In this study, BDNF levels in dementia patients were higher than those in MCI patients and cognitively normal elderly individuals in the total sample and most subsamples. Compared with normal elderly individuals, there were no significant BDNF alterations (higher or lower) in MCI patients in the total sample and most subsamples. From the change trend, BDNF showed three change patterns with cognitive impairment: (1) BDNF decreased in the MCI stage and increased in the dementia stage. This pattern was observed in the total sample (clinical diagnosis and MMSE group) and subsamples (men and women, 51–64 years old and 65–74 years old group, physical and technical occupation, 7–9 years education group, and *APOE4* negative and positive group); (2) BDNF increased gradually with cognitive impairment. This pattern was found in the CDR group, over 75 years old group, 0–6 years and more than 10 years of education group, intellectual occupation, and Shanghai sample; (3) Although the unified training has ensured the consistency of research methods, etc., the trend of BDNF values may be different due to regional differences. Therefore, after analyzing the trends in different centers, this study uses the overall trend to conduct a comparative study. As a result, BDNF increased in the MCI stage and decreased in the dementia stage. This pattern was found in Wuxi, Jiangyi, and Hangzhou samples. The three change patterns are supported by research evidence; for example, Woolley et al. (2012) and Forlenza et al. (2015) supported the pattern with BDNF decreasing early and subsequently increasing; Sonali et al. (2013) and Lee et al. (2015) studies supported the gradually increasing BDNF pattern. The results of Laske et al. (2006), Angelucci et al. (2010), and Faria et al. (2014) support the model in which BDNF first increases and then decreases.

These discrepancies might be explained by the heterogeneity of AD samples (Balietti et al., 2018) and BDNF compensatory mechanisms (Laske et al., 2006). BDNF plays an important role in the pathogenesis of AD (Song et al., 2015); with the progression of AD, brain-derived BDNF gradually decreases, and BDNF alterations in peripheral blood may be more complicated. In the early stage of MCI, peripheral blood BDNF can supplement BDNF deficiency in the brain through the blood–brain barrier (Pan et al., 1998; Klein et al., 2011), and peripheral blood BDNF may decrease slightly. In the late stage of MCI or early stage of dementia, the continuous decrease in BDNF may stimulate the compensatory mechanism (increased platelet-derived BDNF synthesis and release) (Fujimura et al., 2002), resulting in increased plasma BDNF levels, and in the late stage of dementia, compensatory failure will reduce blood BDNF levels.

A large number of studies suggest that blood BDNF could be used as a biomarker for AD diagnosis, prognosis, and treatment monitoring, but the survey results of blood BDNF levels in AD patients are very inconsistent (Bathina and Das, 2015; Song et al., 2015; Marta et al., 2020; Xie et al., 2020; Mori et al., 2021). There were not only differences in demographic data (such as sex, age and education) and disease status (stage, severity, medication or comorbidity) among different studies but also differences in demographic data between the AD group and the control group in the same study. The heterogeneity of these samples may be an important reason for the inconsistency of research results (Balietti et al., 2018).

Many studies have found that there are differences in blood BDNF levels by sex (women higher than men), age (decrease with age), plasma/serum (serum higher than plasma), disease stage (increase in the early stage and decrease in the late stage), cognitive impairment severity, depressive symptoms, medication and other factors that also affect blood BDNF levels (Lommatzsch et al., 2005; Laske et al., 2006; Fukumoto et al., 2010). In line with these findings, the present study found that plasma BDNF levels were affected by age, education, occupation and sample source, and BDNF differences among clinical diagnosis groups remained after excluding the influence of each covariate.

In addition, demographic variables such as gender, age and education and clinical variables such as AD severity, disease stage and medication cannot only affect blood BDNF alone but also comprehensively affect BDNF through interactive or synergistic mechanisms. This study further examined the comprehensive impact of multiple factors on BDNF by ANCOVA and linear regression analysis. Four-factor analysis found that diagnosis, age and education had a significant impact on BDNF, which could explain only 3–5% of the variance. When the sample source variable was introduced, only the sample source and gender had significant effects on BDNF, and the explained variance reached 38%. It is worth noting that in this study, the sample source is not a simple variable. It includes not only demographic differences such as gender, age, education and occupation but also AD stage and severity, concomitant diseases and medication, blood sample processing and storage duration. These findings remind us that to make BDNF a biomarker of complex diseases such as AD, we must establish standardized detection methods and cut off values of BDNF according to the weight of each influencing factor.

## Data availability statement

The dataset generated during and analyzed during the current study are available from the corresponding author on reasonable request.

## Ethics statement

The studies involving human participants were reviewed and approved by the Wuxi Mental Health Center. The patients/participants provided their written informed consent to participate in this study.

## Author contributions

ZC designed the study, analyzed the data, and revised the manuscript. FQ and HY collected and drafted the manuscript. JL, HZ, and YW participated in the design of the study, data management, and analysis. ZW contributed to data collection management. All authors contributed to the article and approved the submitted version.
